# Efficacy and Safety of Runzao Zhiyang Capsule as an Add-On Therapy for Chronic Eczema: A Systematic Review and Meta-Analysis

**DOI:** 10.1155/2021/6693268

**Published:** 2021-03-17

**Authors:** Ming Li, Boyang Zhou, Lihong Zhou, Linfeng Li

**Affiliations:** Department of Dermatology, Beijing Friendship Hospital, Capital Medical University, Beijing, China

## Abstract

**Background:**

Runzao Zhiyang capsule (RZC), an oral Chinese herbal medicine, has been widely used for chronic eczema in China for many years. This study aims to evaluate the efficacy and safety of RZC as an add-on therapy to conventional treatment for chronic eczema.

**Methods:**

Randomized controlled trials (RCTs) assessing the efficacy and safety of RZC as an add-on therapy for chronic eczema were retrieved from eight literature databases from their inception to 31 August, 2020, including CNKI, WanFang, VIP, Sinomed, PubMed, Cochrane Library, Web of Science, and Embase. The Cochrane risk of bias tool was used to assess the methodological quality of the included studies. The data were analyzed by RevMan5.3 software.

**Results:**

A total of 18 RCTs involving 1896 patients with chronic eczema were included. Compared with no oral treatment, RZC was superior on the total efficacy rate (TER) (RR = 1.45, 95% CI: 1.23 to 1.72, *P* < 0.0001), Eczema Area and Severity Index (EASI) (MD = −0.73, 95% CI: −0.90 to −0.56, *P* < 0.00001), and Visual Analogue Scale (VAS) for pruritus (MD = −2.76, 95%CI: −4.53 to −0.99, *P*=0.002). Similar results were also seen in a randomized, placebo-controlled trial. Compared with the antihistamine (AH) group, TER in the RZC combined with AH group was significantly higher (RR = 1.32, 95% CI: 1.21 to 1.43, *P* < 0.00001), and the EASI score (MD = −0.29, 95% CI: −0.38 to −0.20, *P* < 0.001), the VAS score (MD = −0.19, 95% CI: −0.23 to −0.15, *P* < 0.00001), and the level of serum total IgE (MD = −9.83 ng/ml, 95% CI: −11.66 to −8.00 ng/ml, *P* < 0.00001) decreased more significantly in the RZC combined with AH group. In terms of safety, mild gastrointestinal diseases occurred more frequently in the RZC group, and no serious adverse effect was reported.

**Conclusions:**

RZC as an add-on therapy to conventional treatment shows good effects on chronic eczema, and there is no severe side effect from short-term use of RZC. However, due to suboptimal quality of the included studies, more large-sample and high-quality RCTs are needed to improve the evidence quality.

## 1. Introduction

Eczema, also known as “atopic dermatitis” or “neurodermatitis” in many countries, is a common skin disease characterized by a wide spectrum of skin lesions and pruritus [1, 2]. It affects 15% to 20% of children and 1% to 3% of adults around the world [3]. In China, the prevalence of eczema in children is estimated to be 8.3% to 12.94% [4, 5]. The pathogenesis of eczema has not been fully understood, and it is associated with genetic and environmental factors. Although not life threatening, eczema can lead to sleeplessness and have a substantial impact on quality of life. Patients with eczema are more likely to suffer from anxiety and depression [6, 7], resulting in serious public health problems and economic costs [8].

Currently, topical treatments are the mainstay for the treatment of mild to moderate eczema. Moisturizers are widely applied to restore epidermal barrier function. Topical corticosteroids (TCSs) and topical calcineurin inhibitors (TCIs) could reduce lesions and relieve pruritus by inhibiting skin inflammation. Topical antiseptics showed antimicrobial activity against *Staphylococcus aureus*, which is predominantly associated with flares of eczema. Moreover, antihistamines (AHs) are recommended to be used as a systemic add-on treatment to relieve pruritus [2, 9]. However, the side effects of AH, such as drowsiness and fatigue, may impair concentration and reduce productivity. Moreover, some patients with chronic eczema are not satisfied with the effect of AH combined with topical medicine. In order to avoid adverse effects and to attain better clinical effects, many patients with different dermatological diseases, including chronic eczema, have chosen to use complementary and alternative medicines for the treatment of eczema, such as Chinese herbal medicine (CHM) [10–12].

In China, Runzao Zhiyang capsule (RZC), an oral CHM, has been widely used to treat chronic eczema for many years. It is a mixture of six Chinese herbs, including *Polygoni Multiflori* Radix, *Polygoni Multiflori* Radix Praeparata, *Rehmanniae* Radix, Mori folium, *Sophorae flavescentis* Radix, and *Urtica dentata* Hand. The details of RZC are provided in Table 1. A lot of modern pharmacological studies have proved the effects of these components. *Polygoni Multiflori* Radix shows anti-inflammatory activity by decreasing Th2 cytokine levels, such as interleukin- (IL-) 4, IL-5, and IL-13, and inhibiting the mRNA expression of GATA-3 [13]. *Rehmanniae* Radix could inhibit the mRNA expression of IL-4 and tumor necrosis factor- (TNF-) *α* in lesions and suppress mast cell activations, such as histamine release and production of IL-1*β* and IL-6 [14, 15]. Mori folium shows moderate antibacterial activity against *Staphylococcus aureus* and inhibits NF-*κ*B-mediated inflammatory response, such as IL-1*β* and IL-6 [16, 17]. *Sophorae flavescentis* Radix not only inhibits the mast cell-mediated histamine release and decreases the levels of interferon- (IFN-) *γ* and TNF-*α* in lesions but also significantly inhibits the 5-hydroxytryptamine-induced scratching [18, 19]. Total coumarin, an extraction isolated from *Urtica dentata* Hand, increases the production of IL-10 and transforming growth factor- (TGF-) *β* in dendritic cells and induces the generation of CD4^+^CD25^+^ Treg cells [20]. Some clinical trials have reported that RZC combined with biomedicine could reduce skin lesions and relieve pruritus for patients with chronic eczema [21, 22].

Although many randomized controlled trials (RCTs) on RZC as an add-on treatment for chronic eczema have been conducted, there is no systematic review and meta-analysis to integrate these RCTs. Therefore, this study aims to systematically evaluate the efficacy and safety of RZC as an add-on therapy to conventional treatment for the treatment of chronic eczema.

## 2. Methods

This article was conducted in accordance with the Preferred Reporting Items for Systematic Reviews and Meta-Analyses (PRISMA) statement [23]. The PRISMA checklist is presented in Supplementary Table 1.

### 2.1. Literature Search

The four English databases and four Chinese databases were searched from their inception to 31 August, 2020, including PubMed, Cochrane Library, Web of Science, Embase, China National Knowledge Infrastructure (CNKI, https://www.cnki.net/), WanFang Database (WanFang, http://new.wanfangdata.com.cn/), Chinese Scientific Journal Database (VIP, http://qikan.cqvip.com/), and Chinese Biomedical Database (Sinomed, http://www.sinomed.ac.cn/). The search strategy was as follows: ((“eczema” [MeSH] OR “eczema” [Title/Abstract]) OR (“dermatitis” [MeSH] OR “dermatitis” [Title/Abstract])) AND (“Runzao Zhiyang capsule” [Title/Abstract] OR “Runzaozhiyang capsule” [Title/Abstract] OR “Run Zao Zhi Yang capsule” [Title/Abstract]). In addition, the reference lists of relevant studies were also searched to identify other potentially eligible studies. The research was conducted by two independent authors (ML and BYZ), and any disagreements were resolved by the third author (LHZ).

### 2.2. Inclusion and Exclusion Criteria

The eligible studies were selected based on the following inclusion criteria: (1) RCTs published in English or Chinese; (2) participants were diagnosed with chronic eczema by dermatologists, regardless of age, gender, and disease duration; (3) the experimental group was treated with RZC or RZC combined with AH, while the control group was treated with no oral treatment, placebo, or AH. All participants were treated with the same topical medicine, including moisturizers, TCSs, TCIs, and topical antiseptics. (4) The primary outcomes included total efficacy rate (TER) and Eczema Area and Severity Index (EASI). TER is the proportion of participants with the improvement of symptoms and signs ≥60% of baseline at the end of treatment, and EASI is an international tool to assess overall severity of skin lesion [24]. The secondary outcomes included severity of pruritus, serum total IgE level, and adverse events. The Visual Analogue Scale (VAS) is used to assess the severity of pruritus [25].

Animal experiments, case reports, reviews, duplicate studies, inappropriate interventions, and unavailable studies were excluded.

### 2.3. Study Selection and Data Extraction

Based on the inclusion and exclusion criteria, two authors (ML and BYZ) independently screened the titles and abstracts to identify potentially eligible studies and read full texts to determine the final included studies. The disagreement was settled by the third author (LHZ). The extracted data included the first author, publication year, the number of participants in each group, gender, age, interventions, and outcomes.

### 2.4. Quality Assessment

The methodological quality of the included studies was evaluated using the Cochrane risk of bias tool [26]. It contains seven items, including random sequence generation, allocation concealment, blinding of participants and personnel, blinding of outcome assessment, incomplete outcome data, selective reporting, and other bias. The baselines of disease severity between two groups were considered as the source of other bias. Quality of each item was divided into high, unclear, and low risk of bias. Two authors (ML and BYZ) independently conducted the assessment, and any disagreements were tackled by the third author (LHZ).

### 2.5. Statistical Analysis

RevMan5.3 software was used for data analysis. Dichotomous variables were expressed as risk ratio (RR) with 95% confidence interval (CI), and continuous variables were expressed as mean difference (MD) with 95% CI. Heterogeneity between studies was measured by the chi-square test. A fixed-effect model was applied when *I*^2^ < 50% and *P* > 0.10, and a random-effect model was used if not. If ten or more studies were pooled, a funnel plot was used to assess the potential publication bias. Significant differences were accepted when the *P* value <0.05.

## 3. Results

### 3.1. Search Results

**Figure 1:**
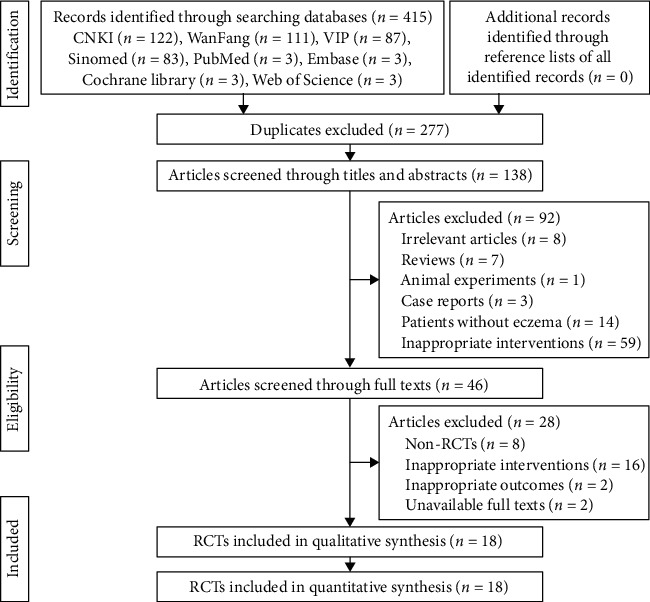
The flowchart of study selection.

A total of 415 studies were identified from eight literature databases, of which 277 duplicates were excluded. 92 studies were excluded based on the titles and abstracts. After screening full texts, 28 studies were excluded further for various reasons. In the end, 18 studies [27–44] met the eligible criteria and were included in this meta-analysis.  Figure 1 shows the flowchart of study selection.

### 3.2. Characteristics of Included Studies

All 18 studies involving 1896 patients were conducted in China. One study published in English was a multicenter clinical trial [33], while the rest were published in Chinese and conducted in a single center. The age of participants ranged from 14 to 79 years old, and the duration range of the treatment was 2 to 8 weeks. Of the included studies, six compared RZC to no oral treatment [27–32], one compared RZC to placebo [33], and eleven compared RZC combined with AH to AH [34–44]. The most frequently used outcome was adverse events, followed by TER, EASI, severity of pruritus, and serum total IgE level. The main characteristics of each study are presented in Table 2.

### 3.3. Quality Evaluation of Included Studies

**Figure 2:**
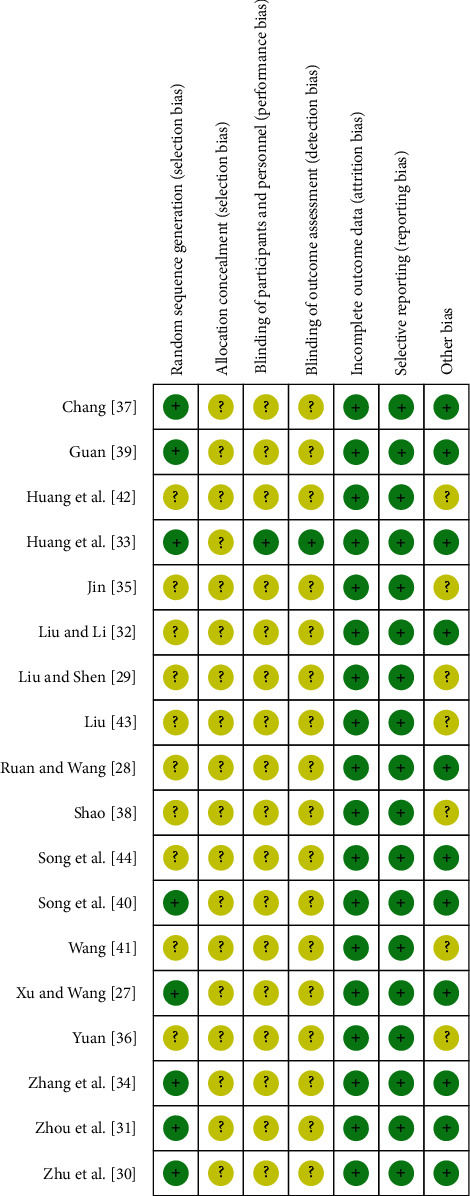
The risk of bias summary of included studies.

Eight studies used random number tables or computer to generate random sequence; therefore, the risk of random sequence generation of them was low [27, 30, 31, 33, 34, 37, 39, 40]. Due to the lack of detailed information, the risk of random sequence generation of the remaining 10 studies was unclear. All studies failed to mention the methods of allocation concealment. Only one study took measures in blinding patients, healthcare providers, and outcome assessors [33]. All studies provided complete outcome data and reported predetermined outcome indicators. Due to the comparability of baselines of disease severity in two groups, other bias was evaluated to be low risk in eleven studies [27, 28, 30–34, 37, 39, 40, 44]. The rest did not report baseline comparability and were considered to be unclear risk.  Figure 2 shows the risk of bias summary of included studies.

### 3.4. Outcomes

#### 3.4.1. Total Efficacy Rate

**Figure 3:**
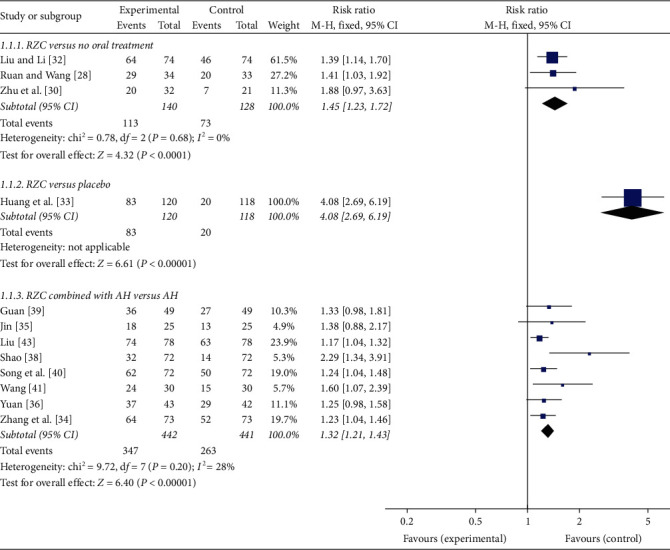
Forest plot of Runzao Zhiyang capsule on the TER.

Three studies reported the TER between the RZC group and the no oral treatment group. No significant heterogeneity was found (*I*^2^ = 0%, *P*=0.68), and a fixed-effect model was used. The pooled results showed that RZC was superior to no oral treatment on the TER (RR = 1.45, 95% CI: 1.23 to 1.72, *P* < 0.0001) [28, 30, 32] (Figure 3).

One randomized, placebo-controlled trial investigated the effect of RZC on chronic eczema. The result revealed that the RZC group had a significantly higher TER compared with the placebo group after 4 weeks of treatment (RR = 4.08, 95% CI: 2.69 to 6.19, *P* < 0.00001) [33] (Figure 3).

Eight studies, which compared RZC combined with AH to AH, used TER as an outcome indicator. Due to low statistical heterogeneity (*I*^2^ = 28%, *P*=0.20), a fixed-effect model was applied. The pooled results showed that the TER of the RZC combined with AH group was significantly higher than that of the AH group (RR = 1.32, 95% CI: 1.21 to 1.43, *P* < 0.00001) [34–36, 38–41, 43] (Figure 3).

#### 3.4.2. EASI

**Figure 4:**
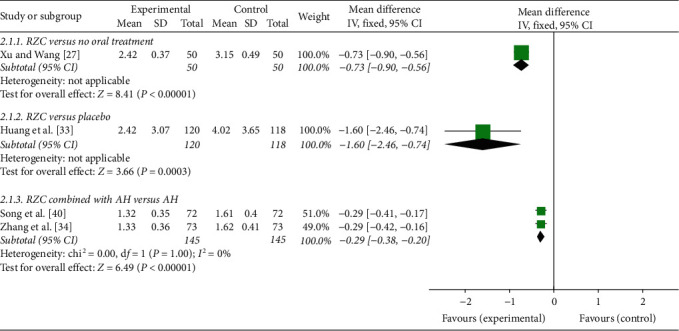
Forest plot of Runzao Zhiyang capsule on the EASI.

One study used EASI to compare the effects between RZC and no oral treatment. The result indicated that RZC was more effective than no oral treatment in terms of reducing EASI score after 4 weeks of treatment (MD = −0.73, 95% CI: −0.90 to −0.56, *P* < 0.00001) [27] (Figure 4).

One randomized, placebo-controlled trial found that the EASI score of the RZC group was significantly lower than that of the placebo group after 4 weeks of treatment (MD = −1.60, 95% CI: −2.46 to −0.74, *P*=0.0003) [33] (Figure 4).

EASI score of the RZC combined with AH group and the AH group was evaluated in two studies [34, 40]. Due to no significant heterogeneity (*I*^2^ = 0%, *P*=1.00), a fixed-effect model was used. The pooled results showed that the EASI score of the RZC combined with AH group could be significantly reduced in comparison with the AH group (MD = −0.29, 95% CI: −0.38 to −0.20, *P* < 0.00001) (Figure 4).

#### 3.4.3. Severity of Pruritus

**Figure 5:**
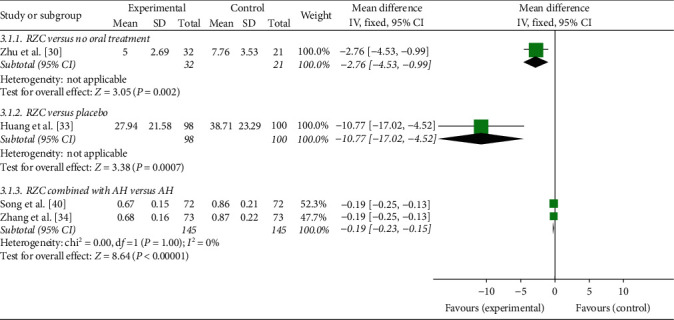
Forest plot of Runzao Zhiyang capsule on the VAS score.

One study, which compared RZC with no oral treatment, found that VAS score of the RZC group was significantly lower than that of the control group after 4 weeks of treatment (MD = −2.76, 95% CI: −4.53 to −0.99, *P*=0.002) [30] (Figure 5).

One randomized, placebo-controlled trial used VAS to evaluate the severity of pruritus during 4 weeks of treatment, and the result revealed that RZC could relieve pruritus in comparison to the placebo (MD = −10.77, 95% CI: −17.02 to −4.52, *P*=0.0007) [33] (Figure 5).

VAS score of the RZC combined with AH group and the AH group was measured in two studies [34, 40]. There was no significant heterogeneity between them (*I*^2^ = 0%, *P*=1.00), and a fixed-effect model was used. The pooled results showed that RZC combined with AH was superior to AH alone in alleviating pruritus (MD = −0.19, 95% CI: −0.23 to −0.15, *P* < 0.00001) (Figure 5).

#### 3.4.4. Serum Total IgE Level

**Figure 6:**
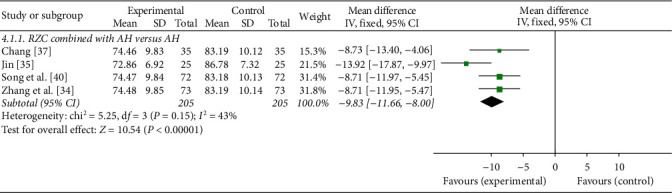
Forest plot of Runzao Zhiyang capsule on the serum total IgE level.

Serum total IgE level was reported in four studies which compared RZC combined with AH to AH alone [34, 35, 37, 40]. Low heterogeneity was observed (*I*^2^ = 43%, *P*=0.15), and a fixed-effect model was used. The pooled results showed that the serum total IgE level of the RZC combined with AH group was significantly lower than that of the AH group (MD = −9.83 ng/ml, 95% CI: −11.66 to −8.00 ng/ml, *P* < 0.00001) (Figure 6).

#### 3.4.5. Adverse Events

**Figure 7:**
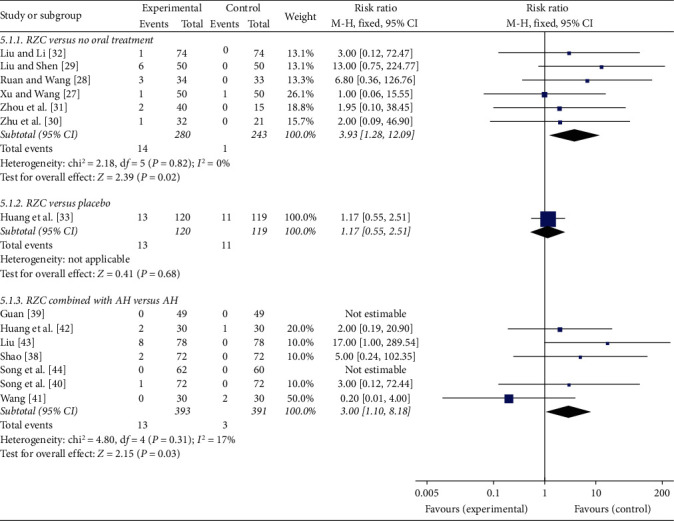
Forest plot of Runzao Zhiyang capsule on the gastrointestinal adverse events.

In total, 14 studies mentioned adverse events during the treatment [27–33, 38–44]. In terms of gastrointestinal adverse events, few patients in the RZC group experienced gastrointestinal diseases, such as mild diarrhea and gastrointestinal discomfort. Six studies reported gastrointestinal adverse events in the RZC group and the no oral treatment group [27–32]. No significant heterogeneity was found (*I*^2^ = 0%, *P*=0.82), and a fixed-effect model was used. The pooled results showed that the incidence of gastrointestinal adverse events of the RZC group was significantly higher than that of the no treatment group (RR = 3.93, 95% CI: 1.28 to 12.09, *P*=0.02) (Figure 7). The same result was also seen in the seven studies comparing RZC combined with AH to AH lone [38–44]. Due to low heterogeneity (*I*^2^ = 17%, *P*=0.31), a fixed-effect model was used. The pooled results showed that RZC combined with AH significantly increased the incidence of gastrointestinal diseases compared with AH alone (RR = 3.00, 95% CI: 1.10 to 8.18, *P*=0.03) (Figure 7). However, the randomized, placebo-controlled trial found that 13 patients in the RZC group and 11 patients in the placebo group suffered from gastrointestinal diseases, and the incidences between two groups were not statistically different (RR = 1.17, 95% CI: 0.55 to 2.51, *P*=0.68) (Figure 7) [33].

In terms of laboratory examinations, six studies monitored blood routine, urine routine, liver function, and renal function during the 2 to 8 weeks of treatment. Five out of the six studies reported that 293 patients in the RZC group had normal results of these laboratory examinations [30, 39, 40, 43, 44]. In the randomized, placebo-controlled trial, 7 patients (7/120, 5.83%) in the RZC group and 11 patients (11/119, 9.24%) in the placebo group experienced a slight increase of liver transaminases, respectively, and the incidences of abnormal liver function between two groups were not significantly different (RR = 0.63, 95% CI: 0.25 to 1.57, *P*=0.32) [33].

### 3.5. Publication Bias

Because there were less than 10 studies in each comparison, the funnel plot was not used to assess the publication bias of the included studies.

## 4. Discussion

To the best of our knowledge, this is the first meta-analysis to assess the efficacy and safety of RZC as an add-on therapy for chronic eczema. The current study showed that the RZC group had a higher TER and a lower EASI score in comparison with the no oral treatment group and the placebo group. The pathogenesis in eczema is complex, and many different cytokines are involved, such as IL-4, IL-5, IL-13, and IL-17. Because of multiple ingredients with immune regulation function, RZC could possibly treat chronic eczema by multiple mechanisms. Some clinical trials have proved that RZC could rapidly decrease the levels of IL-1, IL-4, and TNF-*α* in serum [45, 46]. One study on rats shows that RZC could regulate inflammation by decreasing the levels of IL-6 and IL-17 and increasing the level of IL-10 in serum [47]. Therefore, RZC has a good effect on the treatment of chronic eczema and is effective as an add-on treatment to topical medicine. In addition, the current study also found that compared with AH, RZC combined with AH could significantly improve TER and reduce EASI score, suggesting that RZC is also effective as an add-on treatment to AH and topical medicine. Therefore, the combination of RZC, AH, and topical medicine may provide a new treatment for refractory chronic eczema.

Itch is a defining symptom of eczema. It leads to scratching, resulting in more inflammation of skin, and a vicious “itch-scratch” circle is initiated. The pathophysiology of itch in eczema is not fully understood. Besides mast cell-histamines axis, basophiles are involved in acute itch flares, and many cytokines have also been identified, such as IL-4, IL-13, and substance P [48, 49]. The current study showed that the VAS score of the RZC group reduced significantly compared with the placebo group and the no oral treatment group, indicating that RZC as an oral CHM is able to relieve itch effectively. In addition, the VAS score of the RZC combined with AH group was significantly lower than that of the AH group, suggesting that RZC could alleviate pruritus though histamine-independent pathways. The possible mechanism may be attributed to the fact that RZC could suppress the production of some inflammatory cytokines, such as IL-4 and IL-13. Therefore, RZC is effective as an add-on treatment for relieving itch.

In addition, some laboratory abnormalities can be found in the majority of eczema patients, such as serum total IgE level. IgE is a key molecule which can activate mast cells and basophils in allergic inflammation, and increased serum total IgE level is significantly correlated with the disease severity [50]. The current study showed that the serum total IgE level of the RZC combined with AH group was significantly lower than that of the AH group, which was also consistent with the change of EASI score. The result may be related to the effect of RZC on decreasing the level of IL-4 in serum.

In terms of safety, the current study showed that gastrointestinal diseases were the common adverse events of RZC, such as stomach discomfort and diarrhea, and gastrointestinal adverse events occurred more frequently in patients treated with RZC. Because the symptoms were mild and tolerable, there is no need to discontinue the treatment, and the symptoms could disappear spontaneously after drug withdrawal. On the other hand, laboratory examination is an important aspect of the drug safety. According to the six included studies, the majority of patients in the RZC group had normal liver and renal functions, while a few patients experienced a slight increase of liver transaminases, and the incidences of abnormal liver function between the RZC group and the placebo group were not statistically different [33]. In Chinese literature databases, five cases of liver injury during the treatment of RZC were reported, and the authors claimed the liver dysfunction may be related to *Polygoni Multiflori* Radix, an ingredient of RZC [51–54]. Multiple factors, such as genetic susceptibility and drug dosage, are involved in the mechanism of *Polygoni Multiflori* Radix-induced liver injury [55, 56]. The correlation between RZC and liver injury is still uncertain and more clinical studies are needed. Therefore, administration of routine dose of RZC in the short time is safe, and regular examination of liver and renal function during the treatment can eliminate the occurrence of serious adverse events.

There are some limitations in this meta-analysis. First, the methodological quality of included studies is suboptimal. Most studies did not provide the information on the methods of randomization and blindness, which could reduce the reliability of results. Secondly, the number of the included studies was limited, and the sample sizes were small. Thirdly, the follow-up of the included studies is short. The efficacy and safety of the long-term use of RZC require a larger number of longer follow-up studies in the future. Finally, the conclusions on RZC for chronic eczema could not be generalized to children and other countries. In this study, few participants were children and all included studies were conducted in China.

## 5. Conclusion

In summary, RZC as an add-on therapy to conventional therapy has a good effect on chronic eczema in reducing skin lesion, relieving pruritus, and decreasing serum total IgE level. There does not appear to be any severe side effects from short-term use of RZC, and regular detection of liver and renal function is necessary in case of serious adverse events. However, because the methodological quality of the included studies is suboptimal, more large-scale and high-quality studies are needed to confirm the current results in the future.

## Figures and Tables

**Table 1:** Ingredients of Runzao Zhiyang capsule^*∗*^.

Herb	The part of the plant	Preparation method	Chinese name	The name from Chinese Pharmacopoeia
*Reynoutria multiflora* (Thunb.) Moldenke	Root	Water extract	He-Shou-Wu (何首乌)	*Polygoni multiflori* Radix
*Reynoutria multiflora* (Thunb.) Moldenke	Root	Powder	Zhi-He-Shou-Wu (制何首乌)	*Polygoni multiflori* Radix Praeparata
*Rehmannia glutinosa* (Gaertn.) DC	Root	Water extract	Sheng-Di-Huang (生地黄)	*Rehmanniae* Radix
*Morus alba* L.	Leaf	Water extract	Sang-Ye (桑叶)	Mori folium
*Sophora flavescens* Aiton	Root	Water extract	Ku-Shen (苦参)	*Sophorae flavescentis* Radix
*Laportea bulbifera* (Sieb. et Zucc.) Wedd.	Root	Water extract	Hong-Huo-Ma (红活麻)	*Urtica dentata* Hand

^*∗*^Each RZC is 500 mg and contains 85 mg of *Polygoni multiflori* Radix, 77 mg of *Polygoni multiflori* Radix Praeparata, 125 mg of *Rehmanniae* Radix, 85 mg of Mori folium, 85 mg of *Sophorae flavescentis* Radix, and 43 mg of *Urtica dentata* Hand.

**Table 2:** The characteristics of 18 included studies.

Study ID	Sample size (male/female)		Age (years)		Interventions		Treatment duration (weeks)	Outcomes
	E	C	E	C	E	C		
Xu and Wang [27]	50 (28/22)	50 (29/21)	27–61	27–60	RZC^#^ + IC	Hydrocortisone butyrate ointment	4	②⑤
Huang et al. [33]	120 (58/62)	118 (51/67)	18–70	19–69	RZC^#^ + urea ointment	Placebo^#^ + urea ointment	4	①②③⑤
Zhang et al. [34]	73 (37/36)	73 (42/31)	22–35	23–34	RZC^#^ + IC	Epinastine capsule + hydrocortisone butyrate ointment	4	①②③④
Jin [35]	25	25	19–38	19–38	RZC^#^ + IC	Epinastine capsule + hydrocortisone butyrate ointment	4	①④
Yuan [36]	43 (23/20)	42 (20/22)	35–65	36–66	RZC^#^ + IC	Desloratadine tablet + urea ointment	4	①
Chang [37]	35 (16/19)	35 (17/18)	18–57	19–58	RZC^#^ + IC	Epinastine capsule + hydrocortisone butyrate ointment	4	④
Shao [38]	72 (35/37)	72 (32/40)	17–66	18–65	RZC^#^ + IC	Levocetirizine tablet + halcinonide ointment	8	①⑤
Guan [39]	49 (24/25)	49 (22/27)	19–57	20–58	RZC^#^ + IC	Mizolastine tablet + halometasone cream	2	①⑤
Song et al. [40]	72 (38/34)	72 (40/32)	23–58	21–59	RZC^#^ + IC	Epinastine capsule + hydrocortisone butyrate ointment	4	①②③④⑤
Wang [41]	30 (15/15)	30 (14/16)	18–60	18–60	RZC^#^ + IC	Epinastine capsule + hydrocortisone butyrate ointment	4	①⑤
Huang et al. [42]	30	30	21–53	21–53	RZC^#^ + IC	Levocetirizine tablet + hydrocortisone butyrate cream	8	⑤
Liu [43]	78 (42/36)	78 (44/34)	14–58	16–60	RZC^#^ + IC	Levocetirizine tablet + hydrocortisone ointment	8	①⑤
Song et al. [44]	62 (37/25)	60 (32/28)	18–60	18–60	RZC^#^ + IC	Mizolastine tablet + Halometasone cream	2	⑤
Ruan and Wang [28]	34	33	18–34	18–34	RZC^#^ + IC	Mometasone furoate cream	4	①⑤
Liu and Shen [29]	50 (32/18)	50 (30/20)	18–60	18–60	RZC^#^ + IC	Dexamethasone ointment	4	⑤
Zhu et al. [30]	32 (21/11)	21 (14/7)	18–46	18–47	RZC^#^ + IC	Vaseline cream + Mupirocin ointment	4	①③⑤
Zhou et al. [31]	40	15	18–70	18–70	RZC^#^ + IC	Hydrocortisone and urea cream	4	⑤
Liu and Li [32]	74	74	19–79	19–79	RZC^#^ + IC	Triamcinolone acetonide and urea ointment	3	①⑤

E: experimental group; C: control group; RZC: Runzao Zhiyang capsule; IC: intervention of the control group. ^#^4 capsules each time and three times a day. ① Total efficacy rate; ② Eczema Area and Severity Index; ③ severity of pruritus; ④ serum total IgE level; ⑤ adverse events.

## Data Availability

The data used to support this study are included within this article.

## Supplementary Materials

Supplementary Materials Supplementary Table 1: PRISMA checklist.
